# Safety and Efficacy in Relapsed or Refractory Classic Hodgkin's Lymphoma Treated with PD-1 Inhibitors: A Meta-Analysis of 9 Prospective Clinical Trials

**DOI:** 10.1155/2019/9283860

**Published:** 2019-12-17

**Authors:** Xueyan Zhang, Li Chen, Yawei Zhao, Huiru Yin, He Ma, Miao He

**Affiliations:** ^1^School of Nursing, Jilin University, Changchun, China; ^2^Department of Pharmacology, College of Basic Medical Sciences, Jilin University, Changchun, China; ^3^Department of Anesthesiology, Second Hospital of Jilin University, Changchun, Jilin, China

## Abstract

**Background:**

Classic Hodgkin's lymphoma (cHL) is characterized by the unique biology in which rare Hodgkin-Reed-Sternberg cells propagate an immunosuppressive microenvironment. Checkpoint inhibitors that target the interaction of PD-1 immune checkpoint receptors have demonstrated remarkable activities in various cancers, such as cHL. This study aims to evaluate the safety and efficacy of PD-1 inhibitors in treating relapsed or refractory cHL (rrHL).

**Methods:**

We searched PubMed, MEDLINE, Embase, Cochrane Central Register of Controlled Trials, China National Knowledge Infrastructure, Wanfang, Chinese Biological Medical Literature, and Abstracts of Conference proceedings of annual meetings without any language restrictions to limit language bias (up to January 2019) for prospective clinical trials that evaluate PD-1 inhibitors in treating relapsed or refractory cHL.

**Results:**

A total of 9 prospective clinical trials with 731 patients were included in the meta-analysis. The pooled risks of all-grade and grade ≥3 adverse events (AEs) were 0.86 (95% CI: 0.66–0.98) and 0.21 (95% CI: 0.17–0.24), respectively. The pooled response, complete response, partial response, and stable disease rates were 0.74 (95% CI: 0.70–0.79), 0.24 (95% CI: 0.18–0.34), 0.48 (95% CI: 0.41–0.55), and 0.15 (95% CI: 0.12–0.17), respectively. The pooled 6-month progression-free survival and 1-year overall survival rates were 0.76 (95% CI: 0.72–0.79) and 0.93 (95% CI: 0.90–0.96), correspondingly.

**Conclusions:**

Our meta-analysis suggested that anti-PD1 monoclonal antibodies improve the outcomes of response and survival rates with tolerable AEs in cHL. However, evidence of immune checkpoint inhibitors for patients with cHL remained insufficient. Well-designed randomized controlled trials or at least nonrandomized trials with a control group should be conducted to confirm the findings of this meta-analysis.

## 1. Introduction

Hodgkin's lymphoma (HL) is a lymphatic system cancer and accounts for 10%–15% of all lymphomas, which involve the liver, lung, and bone marrow at different tumor stages [1]. Classic HL (cHL) is the most common type of HL and accounts for approximately 95% of HL cases [2]. At present, 70%–90% of cHL patients treated through standard chemotherapy or chemoradiotherapy have experienced durable remissions. Patients (10%) with advanced-stage HL have not achieved initial remission, and 30% of responding patients has subsequently relapsed [3, 4]. The standard of care for patients with relapsed or refractory cHL is intensive salvage chemotherapy, followed by autologous hematopoietic cell transplantation, which can produce long-term remission in approximately 50% of patients [5]. However, only 55% of the treated patients have been declared free from treatment failure with an 80% survival rate of 3 years [6].

Immune checkpoint inhibitors (ICIs) have unequivocally attracted considerable attention and have been considered a recent major breakthrough in cancer therapy; ICIs act as monoclonal antibodies (mAbs) to inhibitory receptors on T-cells and other immune cells [7, 8]. Programmed death 1 pathway (PD-1/PD-L1) inhibitors as ICIs have been identified, and multiple agents have been developed by impairing the activation of T-cells and enhancing the self-immune response against cancer cells [9, 10]. PD-1 has been expressed on antigen-stimulated T cells with its ligands PD-L1 and PD-L2 to induce downstream T-cell activation and signaling pathway proliferation and promote immunological self-tolerance [11, 12]. PD-1 inhibitors have been approved for use in various melanomas and cancers and have been expected to be applied to different tumor types in the near future [13, 14]. cHL is characterized by the unique biology, in which rare Hodgkin-Reed-Sternberg (RS) cells propagate an immunosuppressive microenvironment [15, 16]. The PD-1 pathway is crucial in the pathogenesis of HL because chromosome 9p24.1 alterations in RS cells result in the overexpression of PD-L1 and PD-L2 [17, 18], and PD-1 is expressed on immune cells in the HL tumor microenvironment [19, 20].

Nivolumab, pembrolizumab, and atezolizumab have been approved by the U.S. Food and Drug Administration in treating various cancers, such as cHL [21–23]. These drugs have been evaluated through clinical trial registration, including the design phase, to identify the biomarkers that predict favorable clinical response and guide the selection of patients with relapsed cHL [24]. Goldkuhle et al. [25] reviewed the benefits and disadvantages of nivolumab in adults with HL, and the results showed that the 6-month progression-free survival (PFS) is between 60% and 86%, and complete response (CR) rates range from 12% to 29%. However, no meta-analysis has evaluated the safety and effectiveness of PD-1 inhibitors in patients with cHL. Therefore, we performed a meta-analysis to investigate the safety and effectiveness of PD-1 inhibitors in cHL patients and overcome the limitations of individual studies, such as small sample size and lack of statistical power.

## 2. Methods

### 2.1. Identification of Studies

We searched and identified all relevant studies through the following electronic databases: PubMed, Embase, Cochrane Central Register of Controlled Trials, China National Knowledge Infrastructure, Wanfang, Chinese Biological Medical Literature, and Abstracts of Conference proceedings of annual meetings (American Society of Clinical Oncology, American Society of Hematology European, and Hematology Association) without any language restrictions to limit the language bias (up to January 2019). We evaluated the reference lists of all identified references for additional relevant publications through manual retrieval. We combined the following search terms: PD-1, nivolumab, pembrolizumab, sintilimab (IBI-308), and HL. After removing duplicate citations and screening the title and abstracts, we downloaded and assessed the full texts in accordance with the following criteria for eligibility. Two reviewers independently evaluated the screened studies for eligibility. Disagreements were adjudicated by a third reviewer. Our meta-analysis was performed and reported on the basis of the PRISMA statement [26].

### 2.2. Eligibility Criteria

The eligibility criteria were described as follows: (1) a confirmed diagnosis of cHL with all subtypes and stages of HL, undergoing first-line treatment, had relapsed or refractory, without restrictions; (2) the study must be a clinical study related to the efficacy or safety of nivolumab, pembrolizumab, and sintilimab (IBI-308) in treating relapsed or refractory cHL; (3) the study had reported any of the following information: response (pooled response [ORR], CR, partial response [PR], and stable disease [16]), overall survival (OS), and PFS rates and adverse events (AEs). The exclusion criteria were described as follows: (1) studies not related to our research topics or not clinical trials; (2) less than 80% of participants had cHL, unless the publications provided subgroup data for cHL; (3) retrospective studies, letters, editorials, and expert opinions; and (4) studies with insufficient data after contacting the authors.

### 2.3. Data Collection

Two reviewers independently performed data extraction and assessed the methodological quality of eligible studies, and any discrepancies were resolved through a discussion with a third reviewer. The following information was extracted: author, title, publication year, study design, clinical trial government number, intervention, number of patients, type of drugs, median age, ORR, CR, PR, SD, PFS, OS, all-grade, and grade ≥3 AEs.

### 2.4. Assessment of Risk Bias

The quality of eligible studies was assessed using Cochrane Collaboration's risk of bias tool, which included random sequence generation, allocation concealment, blinding of participants and personnel, blinding of outcome reporting, incomplete outcome data, selective reporting, and other items [27]. Studies were graded as having low, unclear, and high risks of bias.

### 2.5. Data Synthesis and Analysis

Review Manager 5.3 was used in the risk of bias analysis of our eligible studies. All meta-analyses were processed on R 3.4.3 software with *meta*package and *metaprop module*. *I*^2^ statistics and *Q* test were used to evaluate the heterogeneity among the studies. Heterogeneity was observed among the studies when *I*^2^ > 50% and *p* < 0.05 of the *Q* test, and a random-effect model was used to compute the overall risk. Otherwise, a fixed-effect model was used to compute the pooled estimate of the overall risk. The overall risk of all-grade and grade ≥3 AEs was used to evaluate the safety of PD-1 inhibitors in treating relapsed or refractory cHL. The efficacy of PD-1 inhibitors in treating cHL was evaluated by calculating the overall ORR, CR, PR, SD, PFS, and OS rates with 95% CI based on the data from eligible studies. Subgroup and sensitivity analyses were performed to assess the sources of heterogeneity and recognize the optimum anti-PD1 inhibitors in treating cHL. Publication bias in the included studies was assessed through the funnel plot asymmetry and linear regression test.

## 3. Results

### 3.1. Study Selection and Characteristics of Eligible References

**Figure 1:**
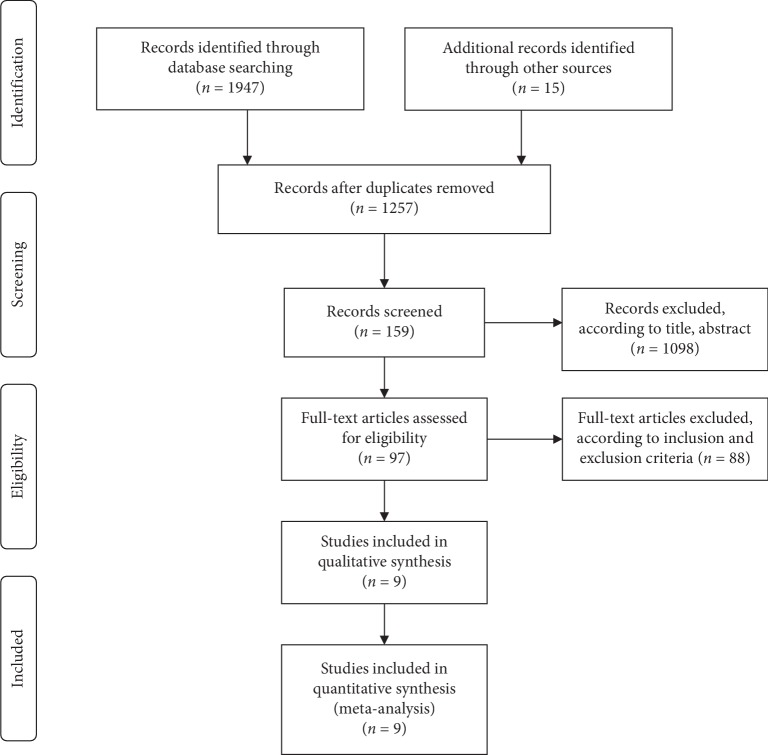
Flowchart of the study selection procedure.

Our literature search strategy yielded 1962 potentially relevant articles. First, 1865 studies were excluded for duplicate articles and eligibility criteria after reviewing titles and abstracts. Second, the remaining 97 articles were full-text screened, and 88 articles were excluded because they did not satisfy the eligibility criteria. Finally, 9 studies [28–36] were considered eligible and included in the meta-analysis (Figure 1). The characteristics of eligible studies are summarized in Table 1. The eligible studies were published from 2015 to 2019; these studies included five phase 1 studies [28–30, 32, 33] and four phase 2 studies [31, 34–36]. All studies were single-arm-designed clinical trials. A total of 731 patients were included in the meta-analysis, in which 282 patients received nivolumab, 241 patients received pembrolizumab, 72 patients received nivolumab + brentuximab vedotin, 31 patients received nivolumab + ipilimumab, and 92 patients received sintilimab (IBI-308). Four of the studies [28, 30, 32, 36] showed incomplete information on response rates (without SD or PFS or OS), and three studies [30, 32, 34] presented incomplete information on AEs.

### 3.2. Safety Analysis

**Figure 2:**
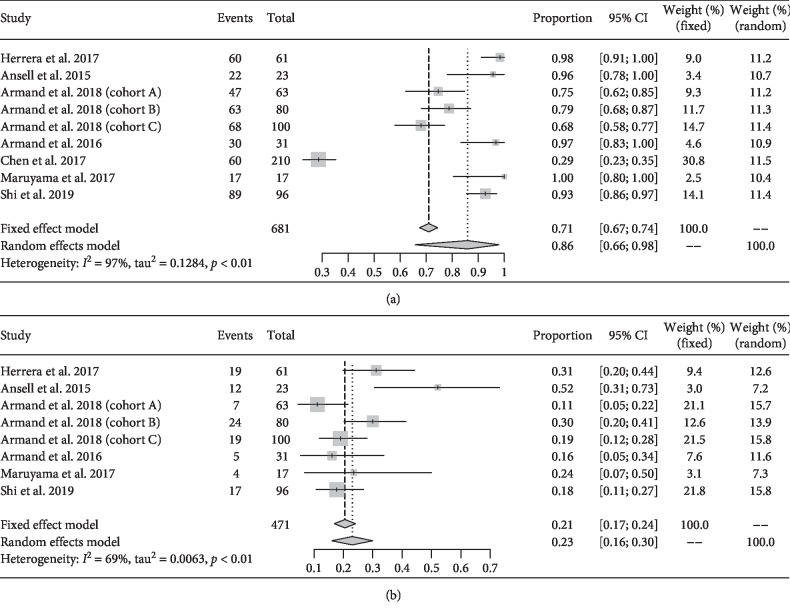
Overall risk of all-grade (a) and grade ≥3 (b) AEs of PD-1 inhibitors in treating relapsed or refractory cHL.

Among all studies, nine data points were included in analyzing all-grade AEs, and eight data points were included in analyzing grade ≥3 AEs. Moreover, other studies with insufficient data were excluded from the analysis. The pooled risks of all-grade and grade ≥3 AEs were 0.86 (95% CI: 0.66–0.98; *I*^2^ = 97.0) and 0.21 (95% CI: 0.17–0.24; *I*^2^ = 69.0), respectively (Table 2, Figure 2). Treatment-related AEs dispersedly occurred on multiple systems, and the majority of individual AEs had low-pooled risks. Peripheral sensory neuropathy was the most common AE with the highest rate of 0.32 (95% CI: 0.05–0.80; *I*^2^ = 88.0). Other common individual AEs were pyrexia (0.28), headache (0.20), fatigue (0.18), nausea (0.18), rash (0.18), infusion-related reactions (0.17), pruritus (0.17), hypothyroidism (0.16), cough (0.15), diarrhea (0.15), and blurred vision (0.15). The rest of all-grade AEs rarely occurred (Table 2). Although grade ≥3 AEs were observed in multiple systems, the rates of the majority of AEs were relatively low [28, 29, 31–36]. The common grade ≥3 AEs were dyspnea (0.1), hypoxia (0.1), pneumonia (0.1), pruritus (0.1), typhlitis (0.1), hyponatremia (0.06), and endocrine disorders (0.6) [32, 35].

### 3.3. Efficacy Analysis

**Figure 3:**
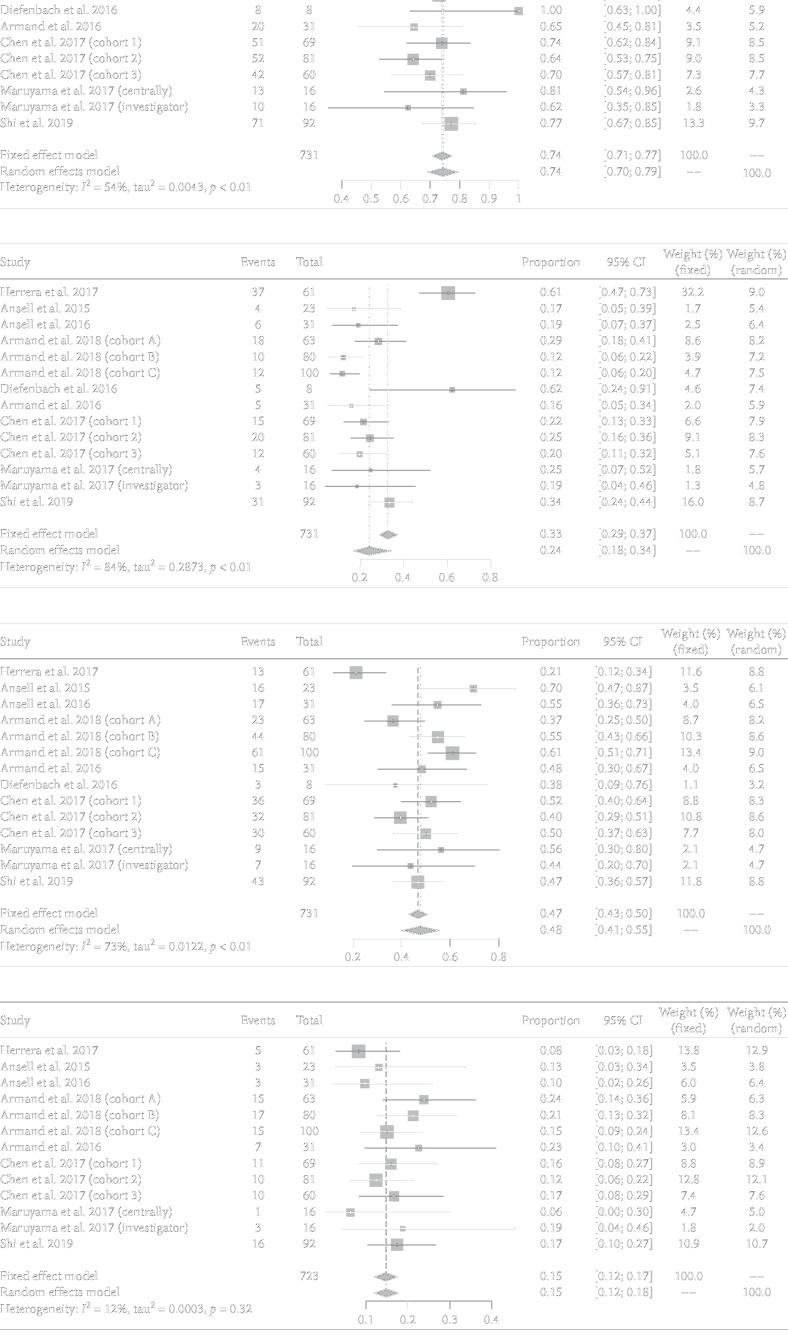
Pooled ORR (a), CR (b), PR (c), and SD (d) rates of PD-1 inhibitors in treating relapsed or refractory cHL.

**Figure 4:**
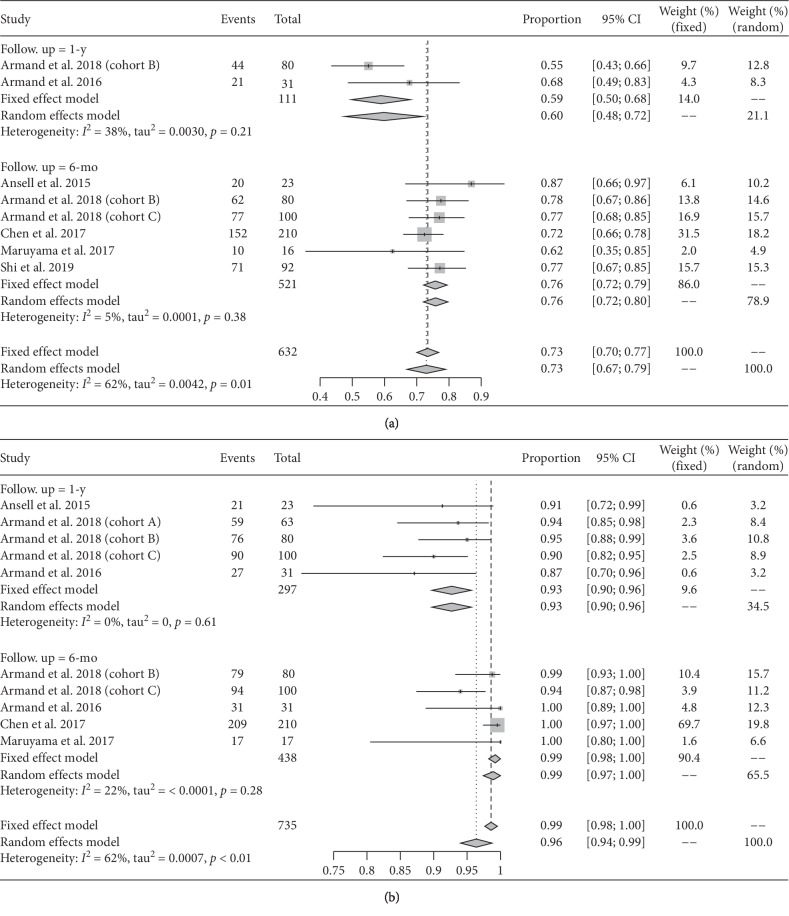
Pooled 1-year and 6-month PFS (a) and OS (b) rates of PD-1 inhibitors in treating relapsed or refractory cHL.

A total of 14 data points were included in analyzing ORR, CR, and PR rates, 13 in analyzing SD rate, 6 in 6-month and 2 in 1-year PFS rate analyses, and 5 in 6-month and 5 in 1-year OS rate analyses. The pooled ORR, CR, PR, and SD rates were 0.74 (95% CI: 0.70–0.79; *I*^2^ = 54.0), 0.24 (95% CI: 0.18–0.34; *I*^2^ = 84.0), 0.48 (95% CI: 0.41–0.55; *I*^2^ = 73.0), and 0.15 (95% CI: 0.12–0.17; *I*^2^ = 12.0), correspondingly (Figure 3). The pooled 6-month PFS and 1-year OS rates were 0.76 (95% CI: 0.72–0.79; *I*^2^ = 5.0) and 0.93 (95% CI: 0.90–0.96; *I*^2^ = 0.0) (Table 3, Figure 4), respectively.

### 3.4. Subgroup and Sensitivity Analyses

The pooled risks of all-grade AEs were 0.87 (95% CI: 0.49–1.00; *I*^2^ = 98.0) with 35 or older patients, which had no significant differences with patients younger than 35 years (0.85, 95% CI: 0.69–0.96; *I*^2^ = 90.0). The pooled risks were higher in grade ≥3 AEs with 35 or older patients (0.32, 95% CI: 0.49–1.00; *I*^2^ = 98.0) than in patients younger than 35 years (0.16, 95% CI: 0.12–0.20; *I*^2^ = 0.0). The pooled risks were lower in all-grade/grade ≥3 AEs with PD-1 inhibitor monotherapy than in combination therapy. The pooled risks of all-grade/grade ≥3 AEs were higher with nivolumab (0.85/0.25) than with pembrolizumab (0.68/0.16). The pooled risks of AEs were lower in the phase 2 subgroup than in phase 1. No significant differences were observed at the risks of AEs in the prior treatments of patients (Supplementary data: [Supplementary-material supplementary-material-1]).

The pooled ORR, CR, PR, SD, PFS, and OS rates had slight differences between the age and prior treatments of patients. The pooled ORR and CR rates were lower in monotherapy subgroups than in combination therapy, whereas the PR and SD rates were high in the monotherapy subgroups. As for anti-PD-1 monotherapy, overall response rate was 72%, 69%, and 77% for nivolumab, pembrolizumab, and sintilimab, respectively. Overall response rate in patients who did not receive BV + ASCT was 75%, which was similar in pretreated patients who received ASCT/BV treatments. The pooled ORR, CR, and SD rates were lower in phase 2 than in phase 1, whereas the pooled PR rate was higher in phase 2 (0.49, 95% CI: 0.45–0.53; *I*^2^ = 48.0) than in phase 1 (0.46, 95% CI: 0.26–0.66; *I*^2^ = 84.0). The pooled 6-month PFS rates of nivolumab and pembrolizumab were 0.77 (95% CI: 0.71–0.82; *I*^2^ = 0.0) and 0.72 (95% CI: 0.66–0.78), correspondingly. The pooled 1-year OS rates of nivolumab and pembrolizumab were 0.93 (95% CI: 0.90–0.96; *I*^2^ = 0.0) and 0.87 (95% CI: 0.75–0.99), respectively. All the results of the subgroup analysis in the meta-analysis are presented in the Supplementary data: [Supplementary-material supplementary-material-1].

Sensitivity analyses were performed to evaluate the stability of our results. The results demonstrated that, by removing one study every time, no individual study significantly affected the pooled results, thereby suggesting that our results are credible.

### 3.5. Quality Assessment and Publication Bias of Studies

**Figure 5:**
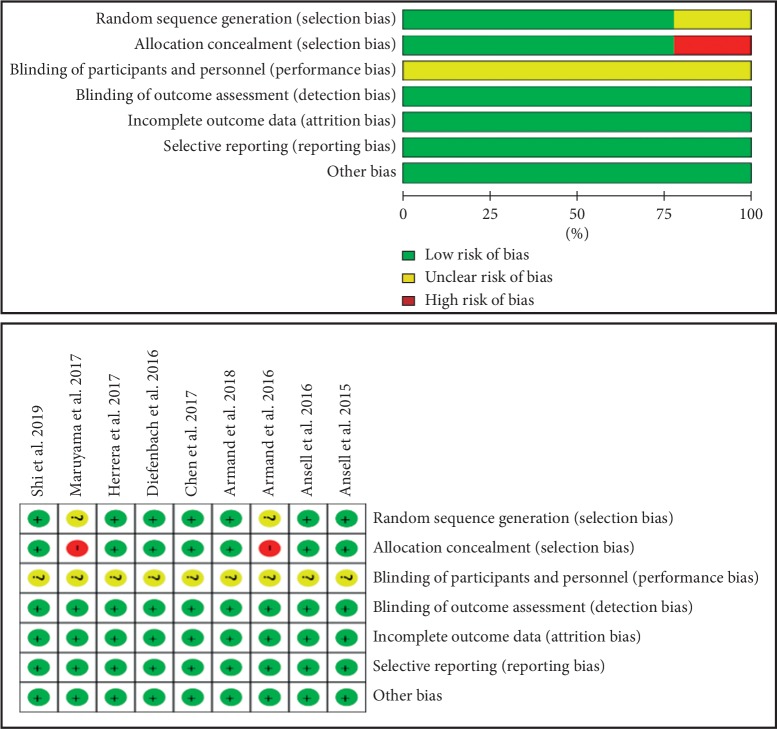
Risk of bias graph and risk of bias summary. Blinding of participants and personnel was not evaluated because all included studies are single-arm-designed trials.

The risk of bias of all included studies is exhibited in Figure 5. The majority of the included studies were randomized design, and blinding of participants and personnel was not evaluated because all included studies were single-arm-designed trials. Therefore, the overall risk of bias was evaluated as low risk, and the quality of eligible studies was satisfactory.

Potential publication bias was observed in the pooled all-grade AEs, CR, and OS rates in the meta-analysis (*p*=0.012, *p*=0.004, and *p*=0.008). After using a trim-and-fill method, no trimming was performed, and the pooled results remained constant in the study (Supplementary data: Figures [Supplementary-material supplementary-material-1]–[Supplementary-material supplementary-material-1]).

## 4. Discussion

ICIs have demonstrated remarkable activities in various malignancies and cancers and have been approved for use in melanoma, non-small-cell lung cancer, renal cell carcinoma, bladder cancer, and squamous cell carcinoma of the head and neck [37]. Previous studies have suggested that ICIs targeting specific immune checkpoint improve the potential of cancer immunotherapy with a long-lasting antitumor response in different cancer patients [13]. Anti-PD-1 antibodies as high selectivity for immunosuppressive inhibitory T-cell receptor exhibit high antitumor activity and low adverse effects, given their extensive specificity for tumor antigen-specific T-cells and small effects on autoreactive T-cells [38]. Data are limited to make a clear statement on anti-PD-1 antibodies for patients with relapsed or refractory cHL, except for heavily pretreated people who had undergone regimens of BV or ASCT previously. Based on overall response and response duration, nivolumab demonstrated a clinically meaningful activity in patients with cHL after the failure of autologous HSCT and post-transplantation BV with an overall favorable benefit-risk balance [39]. This finding prompted us to perform this meta-analysis for evaluating the safety and efficacy of PD-1 inhibitors in relapsed or refractory cHL.

To the best of our knowledge, this comprehensive meta-analysis with existing prospective clinical trials was the first to evaluate the safety and efficacy of PD-1 inhibitors in treating relapsed or refractory cHL. Our meta-analysis results confirmed the favorable safety profile and good toleration to anti-PD-1 inhibitor in rrHL patients. AEs were mainly grade 1 or grade 2 and manageable, and the rate of AEs was similar to that in trials of anti-PD-1 inhibitor in solid tumor patients [29]. The common individual AEs were fatigue, diarrhea, infusion reactions, rash, and grades 3/4 drug-related AEs in more than 3% of the participants including the increase in lipase, alanine aminotransferase, and neutropenia [25]. However, due to fewer clinical trials, small sizes, and the relatively short follow-up times, larger and long-term follow-up trials are needed to confirm the safety of PD-1 inhibitors in rrHL. Nevertheless, the extended analysis after an 18-month follow-up in CheckMate 205 trial presented that safety profile of anti-PD-1 inhibitors remained consistent with previous reports, regardless of patients who received BV before and/or after auto-HCT and patients refractory to previous therapy [31].

Our results showed that PD-1 inhibitor demonstrated high response rates and prolonged survival for rrHL patients, which were similar to that in trials of PD-1 inhibitor in patients with advanced or refractory cancers [40]. As for anti-PD-1 monotherapy, our results demonstrated that nivolumab was associated with an overall response rate of 72%, pembrolizumab of 69%, and sintilimab of 77%, respectively. Clinical trials of nivolumab, pembrolizumab, and sintilimab contribute to the increasing evidence of the role of PD-1 inhibitor in cHL. As for nivolumab in first-line for patients with rrHL, nivolumab was associated with an overall response rate of 65% to 80% in two previous trials, which was similar to our results that an overall response rate of 75% in patients first-line of anti-PD-1 antibodies therapy. Actually, the response rates were similar in patients who received BV after or only before auto-HCT and in patient refractory to their first or last line of therapy or to BV given after auto-HCT [29, 31]. Most rrHL patients of eligible trials in our study received previous therapy, the majority of heavily pretreated patients had a relapse after ASCT and/or BV treatments [29]. Considering the limited disease progresses after ASCT and the relatively short-lived response to BV after relapse, PD-1 inhibitor may represent a promising targeted treatment for patients with rrHL. Nivolumab may have a favorable safety profile and provide long-term benefits to a broad spectrum of patients with rrHL after autologous hematopoietic cell transplantation (auto-HCT) and/or BV treatments [29, 31]. Moreover, Mauyama et al. [35] confirmed the efficacy and safety of nivolumab in Japanese patients with pretreated rrHL after BV, which was also effective with a variety of cHL subtypes. Besides, nivolumab has been approved for the treatment of adults with relapsed/progressed/refractory cHL after auto-HCT and BV treatment by U.S. Food and Drug Administration and European Medicines Agency [31]. In phase Ib and phase II trials, pembrolizumab also provided favorable safety profile and high response rates in patients with pretreated rrHL after ASCT/BV failure [33, 34]. Additionally, sintilimab had favorable activity and safety profile in Chinese patients with rrHL in the phase II, single-arm trial by Shi et al. [36], and all subgroups regardless of they were refractory to first-line/the last previous chemotherapy or the different baseline characteristics had similar benefit from it; that was consistent with the studies of nivolumab and pembrolizumab [34, 41]. In recent years, Goldkuhle et al. [25] performed a meta-analysis to evaluate the benefits and disadvantages of nivolumab in adults with cHL. Their analysis, including three published nonrandomized, uncontrolled trials, revealed that more than 50% of patients who had previously undergone regimens of BV or ASCT with a limited life expectancy were alive from 16 to 23 months, and their CR rates ranged from 12% to 29%. Moreover, serious AEs rarely occurred. Well-designed randomized controlled trials or at least nonrandomized trials with a control group should be conducted to verify the results.

The subgroup analysis results showed that anti-PD-1 antibodies combined with other drugs increase the adverse effect and response rates, which were consistent with the results of many previous individual studies [28, 30, 32]. Nivolumab combined with brentuximab or ipilimumab showed a high response rate and low AEs, which might be due to other existing inhibitory receptors that anti-PD-1 antibodies alone did not completely restore the function of antitumor T-cells [42]. We did not compare anti-PD-1 mAbs with other drugs in cHL because the studies were single-arm-designed clinical trials. In these clinical trials, anti-PD-1 antibodies improved the overall response rates and prolonged OS for patients with cHL. Physicians should balance the clinical outcome and adverse effects when using combination strategies of anti-PD-1 antibodies.

Significant heterogeneity was observed among the eligible studies. Thus, we performed subgroup and sensitivity analyses to investigate the source of heterogeneity. Potential sources of heterogeneity were due to the study design, intervention treatment, doses of drugs, and clinical phase. A random-effect model was used in the pooled analysis with the existence of heterogeneity, and we used a fixed-effect model to estimate the results of pooled analysis. The results of subgroup and sensitivity analyses indicated that no individual study significantly affects the pooled results, where patients with cHL exhibit improved response rate and prolonged OS rate using anti-PD-1 antibodies. We conducted a funnel plot asymmetry and linear regression test to evaluate the publication bias among eligible studies, and the results showed no publication bias in the meta-analysis.

Our meta-analysis had several limitations. First, the prospectively planned, nonrandomized, and uncontrolled trials exhibited a high risk of bias. No standard instrument existed to assess the risk of bias for this type of trials. Second, intervention time and cycles of patients receiving anti-PD-1 inhibitors, inhibitors, and previous treatments of patients were different among eligible studies, thereby possibly causing some biases to the meta-analysis. Third, the data of some influencing factors of cHL in all relevant studies were few or sparse to be evaluated and discussed.

In conclusion, our meta-analysis suggested that anti-PD1 mAbs improved the outcomes of ORR, CR, PR, SD, OS, and PFS rates with tolerable AEs in cHL. Evidence of ICIs for patients with cHL was insufficient. Well-designed randomized controlled trials or at least nonrandomized trials with a control group should be conducted to confirm the findings of this meta-analysis.

## Figures and Tables

**Table 1:** The characteristics of eligible studies.

Study	Clinical trial	Design	No.	Median age (y, range)	Follow-up time (mo)	Prior treatments (no.)	Drugs	ORR/CR/PR/SD/PFS/OS (%)	All-/≥3 AEs (%)
Herrera et al. [28]	NCT02572167	Nonrandomised, open-label, multicenter, single-arm, phase 1/2	62	36 (18–69)	7.8	No BV + ASCT (62)	Nivolumab + BV	82/61/21/8/NA/NA	98/31

Ansell et al. [29]	NCT01592370 (Cohort 1)	Dose escalation and expansion cohorts, phase 1	23	35 (20–54)	10	Prior BV + ASCT (15); Prior BV (3); No BV + ASCT (5);	Nivolumab	All-87/17/70/13/86(6-mo)/91(1-y) Prior BV + ASCT-87/7/80/13/86/NA Prior BV-100/NA/100/NA/NA/NA No BV + ASCT-80/60/20/20/80/NA	96/52
Ansell et al. [30]	NCT01592370 (Cohort 2)		31	35 (20–54)	11.4	Prior ASCT (31)	Nivolumab + IPI	74/19/55/10/NA/NA	NA/NA

Armand et al. [31]	NCT02181738 (Cohort A)	Multicentre, noncomparative, multicohort, single-arm, open-label, phase 2	63	33 (18–65)	18	No BV + ASCT (63)	Nivolumab	65/29/37/24/PFS:77(6-mo); 55(1-y)/OS:99(6-mo); 95(1-y)	75/11
	NCT02181738 (Cohort B)		80	37 (18–72)		Prior BV + ASCT (80)		68/13/55/21/77(6-mo)/OS:93(1-y)	91/30
	NCT02181738 (Cohort C)		100	32 (19–69)		Prior BV + ASCT (100)		73/12/61/15/PFS:77(6-mo)/OS:94(6-mo); 90(1-y)	68/19

Diefenbach et al. [32]	NCT01896999	Multicohort, phase 1	8	46 (25–53)	NA	Prior ASCT/BV (8)	Nivolumab + BV	100/62/23/NA/NA/NA	NA/NA

Armand et al. [33]	NCT01953692	Multicohort, open-label, single-arm, phase 1b	31	32 (20–67)	17	Prior BV + ASCT (31)	Pembrolizumab	65/16/48/23/69(1-y)/87(1-y)	97/16

Chen et al. [34]	NCT02453594 (Cohort 1)	Multicenter, single-arm, phase 2	69	34 (19–64)	10.1	Prior BV + ASCT (69)	Pembrolizumab	74/22/52/16/72(6-mo)/99.5(6-mo)	29/NA
	NCT02453594 (Cohort 2)		81	40 (20–76)		Prior BV (81)		64/25/40/12/72(6-mo)/99.5(6-mo)	
	NCT02453594 (Cohort 3)		60	32 (18–73)		Prior ASCT (60)		70/20/50/17/72(6-mo)/99.5(6-mo)	

Maruyama et al. [35]	JapicCTI-142755	Nonrandomised, open-label, multicentre phase 2	17	63 (29–83)	9.8	Prior BV (17)	Nivolumab	a: 81/25/56/6/60(6-mo)/100(6-mo) b: 63/19/44/19/60(6-mo)/100(6-mo)	100/25

Shi et al. [36]	NCT03114683	Single-arm, open-label, multicenter, phase 2	92	33 (28–43)	10.5	Prior ASCT/BV (92)	Sintilimab	80.4/34/47/17/77.6(6-mo)/NA	93/18

Notes: a: centrally assessed; b: investigator assessed; BV: brentuximab vedotin; ASCT: autologous stem-cell transplantation; IPI: ipilimumab.

**Table 2:** The pooled AEs incidence in all-grade or grade ≥3 or individual.

AEs	Data points	No.	Event rate	95% CI	Heterogeneity	
					*I * ^2^ (%)	*p* for *I*^2^
All-grade	9	681	0.86	0.66–0.98	97.0	<0.01
Grade ≥ 3	8	471	0.21	0.17–0.24	69.0	<0.01
Individual AEs						
General disorders						
Asthenia	2	241	0.03	0.01–0.06	64.0	0.09
Back pain	3	470	0.07	0.02–0.22	85.0	<0.01
Blurred vision	2	27	0.15	0.06–0.34	0.0	0.56
Fatigue	7	615	0.18	0.09–0.31	89.0	<0.01
Infusion-related reactions (IRRs)	4	417	0.17	0.07–0.36	91.0	<0.01
Nasopharyngitis	3	470	0.08	0.01–0.34	90.0	<0.01
Oropharyngeal pain	2	453	0.10	0.08–0.13	57.0	0.13
Pyrexia	6	650	0.28	0.22–0.36	66.0	0.01
Upper respiratory tract infection	5	576	0.07	0.02–0.22	89.0	<0.01
Gastrointestinal disorders						
Abdominal pain	2	304	0.14	0.11–0.19	0.0	0.94
Constipation	5	562	0.08	0.04–0.16	74.0	<0.01
Diarrhea	8	691	0.15	0.08–0.28	88.0	<0.01
Nausea	6	598	0.18	0.09–0.33	90.0	<0.01
Vomiting	4	545	0.11	0.05–0.24	88.0	<0.01
Skin disorders						
Myalgia	5	541	0.10	0.05–0.18	74.0	<0.01
Nasal congestion	3	514	0.08	0.03–0.21	89.0	<0.01
Rash	8	690	0.18	0.13–0.25	69.0	<0.01
Pruritus	6	564	0.17	0.09–0.31	85.0	<0.01
Hepatic disorders						
ALT level increased	2	274	0.08	0.05–0.11	0.0	0.79
AST level increased	2	274	0.07	0.05–0.11	0.0	0.85
Hepatic function abnormal	2	113	0.05	0.02–0.12	55.0	0.14
Lipase level increased	2	119	0.07	0.03–0.13	0.0	0.68
Thyroid disorders						
Hypothyroidism	5	410	0.16	0.09–0.26	72.0	<0.01
Thyroiditis	2	274	0.02	0.00–0.16	77.0	0.04
Musculoskeletal disorders						
Arthralgia	3	514	0.10	0.04–0.24	89.0	<0.01
Respiratory disorders						
Chills	3	302	0.07	0.02–0.23	85.0	<0.01
Cough	4	537	0.15	0.05–0.36	94.0	<0.01
Dyspnea	5	555	0.09	0.04–0.19	80.0	<0.01
Pneumonia	4	380	0.11	0.08–0.15	0.0	0.99
Nervous system disorders						
Dizziness	2	78	0.13	0.07–0.22	0.0	0.88
Headache	3	321	0.20	0.16–0.25	0.0	0.58
Peripheral sensory neuropathy	2	71	0.32	0.05–0.80	88.0	<0.01
Blood and lymphatic system disorders						
Alanine aminotransferase increased	3	349	0.14	0.03–0.45	92.0	<0.01
Alkaline phosphatase increased	4	380	0.06	0.04–0.09	31.0	0.22
Anemia	2	306	0.09	0.06–0.12	0.0	0.61
Aspartate aminotransferase increased	3	349	0.09	0.02–0.37	90.0	<0.01
Decreased lymphocyte count	5	701	0.05	0.04–0.07	0.0	0.72
Decreased platelet count	2	119	0.13	0.08–0.20	0.0	0.44

**Table 3:** The pooled response rate.

		Data points	No.	Event rate	95% CI	Heterogeneity	
						*I * ^2^ (%)	*p* for *I*^2^
ORR		14	731	0.74	0.70–0.79	54.0	<0.01
CR		14	731	0.24	0.18–0.34	84.0	<0.01
PR		14	731	0.48	0.41–0.55	73.0	<0.01
SD		13	723	0.15	0.12–0.17	12.0	0.32

PFS	6-mo	6	521	0.76	0.72–0.79	5.0	0.38
	1-y	2	111	0.59	0.50–0.68	38.0	0.21

OS	6-mo	5	438	0.99	0.98–1.00	22.0	0.28
	1-y	5	297	0.93	0.90–0.96	0.0	0.61

## Supplementary Materials

Supplementary Materials Table S. Subgroup analysis of response rate and AEs incidence in all-grade or grade ≥3 Figure S1. Funnel plot of pooled all-grade AEs (trim-fill method). Figure S2. Funnel plot of pooled CR rate (trim-fill method). Figure S3. Funnel plot of pooled OS rate (trim-fill method).
