# Supplementary material for: Safety and Efficacy in Relapsed or Refractory Classic Hodgkin's Lymphoma Treated with PD-1 Inhibitors: A Meta-Analysis of 9 Prospective Clinical Trials
Source: Biomed Res Int. 2019 Dec 17;2019:9283860. doi: 10.1155/2019/9283860 (PMC6948280; doi:10.1155/2019/9283860)
Supplement: Supplementary Materials — Table S. Subgroup analysis of response rate and AEs incidence in all-grade or grade ≥3 Figure S1. Funnel plot of pooled all-grade AEs (trim-fill method). Figure S2. Funnel plot of pooled CR rate (trim-fill method). Figure S3. Funnel plot of pooled OS rate (trim-fill method). [file 9283860.f1.docx]

Table S. Subgroup analysis of Response rate and AEs incidence in all-grade or grade≥3

|  | Data points | N  fsubjects | Event rate | 95%CI | *I^2^(*%*)* | *P* *for I^2^* |
| --- | --- | --- | --- | --- | --- | --- |
| All-grade AEs |  |  |  |  |  |  |
| Median age |  |  |  |  |  |  |
| ≥35 | 5 | 391 | 0.87 | 0.49-1.00 | 98.0 | <0.01 |
| <35 | 4 | 290 | 0.85 | 0.69-0.96 | 90.0 | <0.01 |
| Therapy |  |  |  |  |  |  |
| Monotherapy | 8 | 620 | 0.84 | 0.62-0.97 | 97.0 | <0.01 |
| Combination | 1 | 61 | 0.98 | 0.94-1.00 | -- | -- |
| Drugs |  |  |  |  |  |  |
| Sintilimab | 1 | 96 | 0.93 | 0.87-0.97 | -- | -- |
| Nivolumab | 5 | 283 | 0.85 | 0.72-0.95 | 86.0 | <0.01 |
| Nivolumab+BV | 1 | 61 | 0.98 | 0.94-1.00 | -- | -- |
| Pembrolizumab | 2 | 241 | 0.68 | 0.03-1.00 | 99.0 | <0.01 |
| Phase |  |  |  |  |  |  |
| Phase=1 | 3 | 115 | 0.97 | 0.94-1.00 | 0.0 | 0.77 |
| Phase=2 | 6 | 566 | 0.78 | 0.52-0.95 | 98.0 | <0.01 |
| Prior treatments |  |  |  |  |  |  |
| No BV+ASCT | 2 | 124 | 0.90 | 0.56-1.00 | 95.0 | <0.01 |
| Prior ASCT/BV | 2 | 113 | 0.97 | 0.83-1.00 | 77.9 | 0.04 |
| Prior BV+ASCT | 5 | 681 | 0.86 | 0.66-0.98 | 97.0 | <0.01 |
| Grade ≥ 3 AEs |  |  |  |  |  |  |
| Median age |  |  |  |  |  |  |
| ≥35 | 4 | 181 | 0.32 | 0.25-0.39 | 35.0 | 0.20 |
| <35 | 4 | 290 | 0.16 | 0.12-0.20 | 0.0 | 0.51 |
| Therapy |  |  |  |  |  |  |
| Monotherapy | 7 | 410 | 0.22 | 0.15-0.29 | 69.0 | <0.01 |
| Combination | 1 | 61 | 0.31 | 0.20-0.43 | -- | -- |
| Drugs |  |  |  |  |  |  |
| Sintilimab | 1 | 96 | 0.18 | 0.10-0.25 | -- | -- |
| Nivolumab | 5 | 283 | 0.25 | 0.14-0.36 | 81.0 | <0.01 |
| Nivolumab+BV | 1 | 61 | 0.31 | 0.20-0.43 | -- | -- |
| Pembrolizumab | 1 | 31 | 0.16 | 0.03-0.29 | -- | -- |
| Phase |  |  |  |  |  |  |
| Phase=1 | 3 | 115 | 0.32 | 0.14-0.49 | 77.0 | 0.01 |
| Phase=2 | 5 | 356 | 0.18 | 0.14-0.22 | 71.0 | 0.07 |
| Prior treatments |  |  |  |  |  |  |
| No BV+ASCT | 2 | 124 | 0.20 | 0.07-0.46 | 86.0 | <0.01 |
| Prior ASCT/BV | 2 | 113 | 0.19 | 0.12-0.27 | 0.0 | 0.57 |
| Prior BV+ASCT | 4 | 234 | 0.27 | 0.17-0.42 | 75.0 | <0.01 |
| ORR |  |  |  |  |  |  |
| Median age |  |  |  |  |  |  |
| ≥35 | 8 | 316 | 0.77 | 0.69-0.86 | 69.0 | <0.01 |
| <35 | 6 | 415 | 0.72 | 0.68-0.76 | 0.0 | 0.58 |
| Therapy |  |  |  |  |  |  |
| Monotherapy | 11 | 631 | 0.72 | 0.68-0.75 | 21.0 | 0.24 |
| Combination | 3 | 100 | 0.85 | 0.72-0.98 | 67.4 | 0.05 |
| Drugs |  |  |  |  |  |  |
| Sintilimab | 1 | 92 | 0.77 | 0.69-0.86 | -- | -- |
| Nivolumab | 6 | 298 | 0.72 | 0.67-0.77 | 39.0 | 0.15 |
| Nivolumab+BV | 2 | 69 | 0.90 | 0.72-1.00 | 75.0 | 0.05 |
| Nivolumab+Ipilimumab | 1 | 31 | 0.74 | 0.59-0.90 | -- | -- |
| Pembrolizumab | 4 | 241 | 0.69 | 0.63-0.75 | 0.0 | 0.58 |
| Phase |  |  |  |  |  |  |
| Phase=1 | 5 | 154 | 0.82 | 0.72-0.92 | 65.0 | 0.02 |
| Phase=2 | 9 | 577 | 0.71 | 0.67-0.75 | 0.0 | 0.52 |
| Prior treatments |  |  |  |  |  |  |
| No BV+ASCT | 3 | 129 | 0.75 | 0.61-0.88 | 58.0 | 0.09 |
| Prior ASCT | 2 | 91 | 0.72 | 0.62-0.81 | 0.0 | 0,67 |
| Prior BV | 2 | 84 | 0.79 | 0.44-1.00 | 76.0 | 0.04 |
| Prior ASCT/BV | 4 | 132 | 0.81 | 0.74-0.88 | 68.0 | 0.02 |
| Prior BV+ASCT | 5 | 295 | 0.72 | 0.67-0.77 | 10.0 | <0.01 |
| CR |  |  |  |  |  |  |
| Median age |  |  |  |  |  |  |
| ≥35 | 8 | 316 | 0.27 | 0.16-0.44 | 86.0 | <0.01 |
| <35 | 6 | 415 | 0.22 | 0.16-0.30 | 65.0 | 0.01 |
| Therapy |  |  |  |  |  |  |
| Monotherapy | 12 | 631 | 0.21 | 0.17-0.27 | 49.0 | 0.03 |
| Combination | 3 | 100 | 0.45 | 0.25-0.81 | 78.0 | 0.01 |
| Drugs |  |  |  |  |  |  |
| Nivolumab | 6 | 298 | 0.18 | 0.12-0.25 | 45.5 | 0.10 |
| Nivolumab+BV | 2 | 69 | 0.61 | 0.49-0.72 | 0.0 | 0.92 |
| Nivolumab+Ipilimumqb | 1 | 31 | 0.19 | 0.09-0.37 | -- | -- |
| Pembrolizumab | 4 | 241 | 0.22 | 0.17-0.27 | 0.0 | 0.33 |
| Sintilimab(IBI-308) | 1 | 92 | 0.34 | 0.25-0.45 | -- | -- |
| Phase |  |  |  |  |  |  |
| Phase=1 | 5 | 154 | 0.32 | 0.17-0.58 | 83.0 | <0.01 |
| Phase=2 | 9 | 577 | 0.22 | 0.17-0.28 | 57.0 | 0.02 |
| Prior treatments |  |  |  |  |  |  |
| No BV+ASCT | 3 | 129 | 0.46 | 0.27-0.80 | 82.0 | <0.01 |
| Prior ASCT | 2 | 91 | 0.20 | 0.13-0.30 | 0.0 | 0.94 |
| Prior BV | 2 | 84 | 0.25 | 0.17-0.36 | 0.0 | 0.67 |
| Prior ASCT/BV | 4 | 132 | 0.36 | 0.28-0.46 | 54.0 | 0.09 |
| Prior BV+ASCT | 5 | 295 | 0.15 | 0.12-0.20 | 9.0 | 0.36 |
| PR |  |  |  |  |  |  |
| Median age |  |  |  |  |  |  |
| ≥35 | 8 | 339 | 0.48 | 0.36-0.59 | 79.0 | <0.01 |
| <35 | 6 | 392 | 0.49 | 0.41-0.57 | 55.0 | 0.05 |
| Therapy |  |  |  |  |  |  |
| Monotherapy | 11 | 631 | 0.50 | 0.45-0.56 | 49.0 | 0.03 |
| Combination | 3 | 100 | 0.37 | 0.13-0.62 | 81.0 | <0.01 |
| Drugs |  |  |  |  |  |  |
| Nivolumab | 6 | 298 | 0.54 | 0.44-0.64 | 64.0 | 0.02 |
| Nivolumab+BV | 2 | 69 | 0.23 | 0.13-0.33 | 0.0 | 0.37 |
| Nivolumab+Ipilimumqb | 1 | 31 | 0.55 | 0.37-0.72 | -- | -- |
| Pembrolizumab | 4 | 241 | 0.47 | 0.41-0.53 | 0.0 | 0.17 |
| Sintilimab(IBI-308) | 1 | 92 | 0.47 | 0.37-0.57 | -- | -- |
| Phase |  |  |  |  |  |  |
| Phase=1 | 5 | 154 | 0.46 | 0.26-0.66 | 84.0 | <0.01 |
| Phase=2 | 9 | 577 | 0.49 | 0.45-0.53 | 48.0 | 0.05 |
| Prior treatments |  |  |  |  |  |  |
| No BV+ASCT | 3 | 129 | 0.28 | 0.20-0.35 | 47.0 | 0.15 |
| Prior ASCT | 2 | 91 | 0.52 | 0.41-0.62 | 0.0 | 0.66 |
| Prior BV | 2 | 84 | 0.68 | 0.09-1.00 | 92.0 | <0.01 |
| Prior ASCT/BV | 4 | 132 | 0.47 | 0.38-0.55 | 0.0 | 0.82 |
| Prior BV+ASCT | 5 | 295 | 0.58 | 0.52-0.63 | 45.0 | 0.12 |
| SD |  |  |  |  |  |  |
| Median age |  |  |  |  |  |  |
| ≥35 | 7 | 308 | 0.12 | 0.08-0.16 | 13.0 | 0.33 |
| <35 | 6 | 415 | 0.17 | 0.14-0.21 | 0.0 | 0.78 |
| Therapy |  |  |  |  |  |  |
| Monotherapy | 11 | 631 | 0.16 | 0.13-0.19 | 0.0 | 0.60 |
| Combination | 2 | 92 | 0.09 | 0.03-0.14 | 0.0 | 0.82 |
| Drugs |  |  |  |  |  |  |
| Nivolumab | 6 | 298 | 0.17 | 0.12-0.21 | 20.0 | 0.28 |
| Nivolumab+BV | 1 | 61 | 0.08 | 0.01-0.15 | -- | -- |
| Nivolumab+Ipilimumqb | 1 | 31 | 0.10 | 0.01-0.20 | -- | -- |
| Pembrolizumab | 4 | 241 | 0.15 | 0.11-0.20 | 0.0 | 0.64 |
| Sintilimab(IBI-308) | 1 | 92 | 0.17 | 0.10-0.25 | -- | -- |
| Phase |  |  |  |  |  |  |
| Phase=1 | 4 | 146 | 0.11 | 0.06-0.16 | 5.0 | 0.37 |
| Phase=2 | 9 | 577 | 0.16 | 0.13-0.19 | 0.0 | 0.50 |
| Prior treatments |  |  |  |  |  |  |
| No BV+ASCT | 3 | 147 | 0.13 | 0.08-0.18 | 66.0 | 0.05 |
| Prior ASCT | 2 | 91 | 0.14 | 0.07-0.21 | 0.0 | 0.33 |
| Prior BV | 2 | 104 | 0.12 | 0.06-0.19 | 0.0 | 0.93 |
| Prior ASCT/BV | 3 | 124 | 0.15 | 0.08-0.21 | 23.0 | 0.27 |
| Prior BV+ASCT | 5 | 303 | 0.17 | 0.13-0.21 | 0.0 | 0.72 |
| 6-m PFS |  |  |  |  |  |  |
| Median age |  |  |  |  |  |  |
| ≥35 | 5 | 326 | 0.73 | 0.68-0.78 | 0.0 | 0.70 |
| <35 | 2 | 192 | 0.77 | 0.71-0.82 | 0.0 | 0.98 |
| Drugs |  |  |  |  |  |  |
| Nivolumab | 5 | 216 | 0.77 | 0.71-0.82 | 0.0 | 0.82 |
| Pembrolizumab | 1 | 210 | 0.72 | 0.66-0.78 | -- | -- |
| Sintilimab(IBI-308) | 1 | 92 | 0.77 | 0.69-0.86 | -- | -- |
| Prior treatments |  |  |  |  |  |  |
| No BV+ASCT | 1 | 5 | 0.80 | 0.45-1.00 | -- | -- |
| Prior ASCT/BV | 2 | 108 | 0.75 | 0.67-0.84 | 23.0 | 0.25 |
| Prior BV+ASCT | 4 | 405 | 0.75 | 0.71-0.79 | 0.0 | 0.69 |
| 1-y OS |  |  |  |  |  |  |
| Median age |  |  |  |  |  |  |
| ≥35 | 2 | 103 | 0.94 | 0.90-0.99 | 0.0 | 0.56 |
| <35 | 3 | 194 | 0.91 | 0.87-0.95 | 0.0 | 0.53 |
| Drugs |  |  |  |  |  |  |
| Nivolumab | 4 | 266 | 0.93 | 0.90-0.96 | 0.0 | 0.61 |
| Pembrolizumab | 1 | 31 | 0.87 | 0.75-0.99 | -- | -- |
| Prior treatments |  |  |  |  |  |  |
| No BV+ASCT | 1 | 63 | 0.94 | 0.88-1.00 | -- | -- |
| Prior ASCT/BV | 1 | 23 | 0.91 | 0.80-1.00 | -- | -- |
| Prior BV+ASCT | 3 | 211 | 0.92 | 0.89-0.96 | 22.0 | 0.28 |





Figure S1. Funnel plot of pooled all-grade AEs (trim-fill method)





Figure S2. Funnel plot of pooled CR rate (trim-fill method)





Figure S3. Funnel plot of pooled OS rate (trim-fill method)
